# The Small Yeast GTPase Rho5 and Its Dimeric GEF Dck1/Lmo1 Respond to Glucose Starvation

**DOI:** 10.3390/ijms19082186

**Published:** 2018-07-26

**Authors:** Hans-Peter Schmitz, Arne Jendretzki, Carolin Sterk, Jürgen J. Heinisch

**Affiliations:** Fachbereich Biologie/Chemie, Universität Osnabrück, AG Genetik, Barbarastr. 11, D-49076 Osnabrück, Germany; Hans-Peter.Schmitz@biologie.uni-osnabrueck.de (H.-P.S.); arne.jendretzki@gmx.de (A.J.); Carolin.Sterk@biologie.uni-osnabrueck.de (C.S.)

**Keywords:** cytoskeleton, nutrient signaling, mitochondria, general stress response

## Abstract

Rho5 is a small GTPase of *Saccharomyces cerevisiae* and a homolog of mammalian Rac1. The latter regulates glucose metabolism and actin cytoskeleton dynamics, and its misregulation causes cancer and a variety of other diseases. In yeast, Rho5 has been implicated in different signal transduction pathways, governing cell wall integrity and the responses to high medium osmolarity and oxidative stress. It has also been proposed to affect mitophagy and apoptosis. Here, we demonstrate that Rho5 rapidly relocates from the plasma membrane to mitochondria upon glucose starvation, mediated by its dimeric GDP/GTP exchange factor (GEF) Dck1/Lmo1. A function in response to glucose availability is also suggested by synthetic genetic phenotypes of a *rho5* deletion with *gpr1*, *gpa2*, and *sch9* null mutants. On the other hand, the role of mammalian Rac1 in regulating the action cytoskeleton does not seem to be strongly conserved in *S. cerevisiae* Rho5. We propose that Rho5 serves as a central hub in integrating various stress conditions, including a crosstalk with the cAMP/PKA (cyclic AMP activating protein kinase A) and Sch9 branches of glucose signaling pathways.

## 1. Introduction

**Figure 1:**
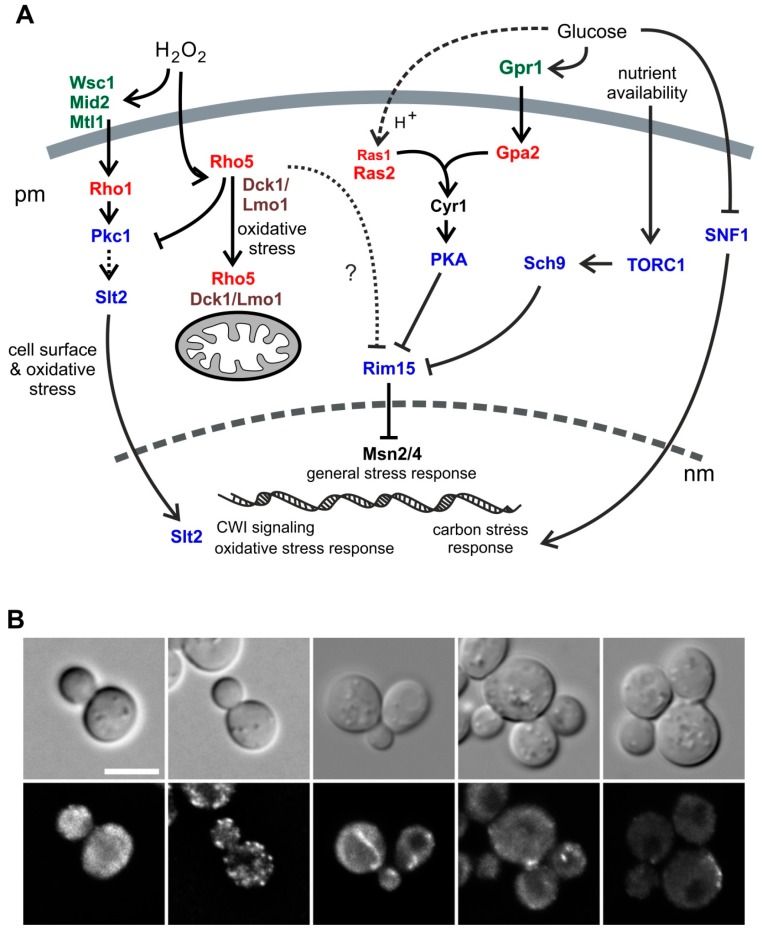
Rho5 in yeast physiology and different distribution patterns of its Dck1 GEF (GDP/GTP exchange factor) subunit. (**A**) Overview on Rho5 and its dimeric GEF in potential interactions with other small GTPases in *S. cerevisiae* discussed in this work. Arrows indicate activation, lines with bars indicate inhibition of the effectors/pathways. pm = plasma membrane, nm = nuclear membrane. (**B**) Examples for alternative distributions of Dck1 in growing yeast cells. The top lane shows bright field images, the lower lane fluorescent images visualizing Dck1-EGFP (GFP optimized for use in eukaryotic cells). Scale bar = 5 µm.

Monomeric G-proteins, also frequently referred to as small GTPases, fulfill essential functions as molecular switches in a variety of biological processes in all eukaryotic cells, including signaling cascades, vesicle trafficking, cytoskeletal organization, and cell migration [1,2]. Among them, the Rho-type GTPases in *Saccharomyces cerevisiae* belong to the Ras superfamily of such proteins, and comprise the essential Rho1 and Cdc42, and the four nonessential members Rho2, Rho3, Rho4, and Rho5 [3]. Rho5 was first described as a regulator of yeast cell wall integrity (CWI) signaling (Figure 1A) [4,5]. Later on, it was also related to HOG (high osmolarity glycerol) signaling [6], with both functions suggested to affect mitophagy and apoptosis [7,8,9]. We recently showed that intracellular distribution of Rho5 is driven by association with its dimeric GDP/GTP exchange factor (GEF) Dck1/Lmo1 under conditions of oxidative stress. Thus, upon addition of hydrogen peroxide the trimeric complex rapidly relocates from a diffuse and partially patchy cytosolic distribution (Dck1/Lmo1; compare Figure 1B) and the plasma membrane (Rho5) to mitochondria, providing a mechanical link to the roles observed in mitophagy and apoptosis [10]. Interestingly, deletion of *RHO5*, and also of *DCK1* or *LMO1*, led to hyper-resistance towards the cell wall stress agents Calcofluor white and Congo red, supporting the originally proposed link to CWI signaling [4,10].

In yeast, glucose signaling is mediated by three complementary routes (reviewed in previous papers [11,12]): (i) cAMP/PKA signaling with two branches involving the small GTPases Ras1/Ras2 and Gpa2, which stimulate cell growth and inhibit stress responses in the presence of glucose, (ii) the SNF1 kinase complex, which represents the yeast homolog of mammalian AMPK, monitors the cells energy state and, in yeast, is activated under glucose-limiting conditions to ensure utilization of alternative carbon sources, and (iii) signaling through the glucose sensors Snf3/Rgt2, which basically regulates the expression of genes encoding glucose transporters. Of those, the SNF1 complex participates in the regulation of cell wall composition, suggested by the sensitivity of null mutants towards cell wall stress agents both in *S. cerevisiae* and in the milk yeast *Kluyveromyces lactis* [13,14]. In addition, Ras2/cAMP-signaling was also found to affect CWI signaling through genetic interactions and in transcriptome studies [15,16], as well as influencing the dynamics of the actin cytoskeleton [17,18], mitochondrial morphogenesis [19], pH homeostasis [20], and apoptosis [21]. Ras/cAMP signaling has also been connected to nutrient signaling by TORC1 through its downstream kinase Sch9 (reviewed in a previous paper [22]; see Figure 1A for an overview of different signaling pathways highlighting the small GTPases involved). Sch9 has been proposed to have a central role in mediating intracellular pH homeostasis [23]. Crosstalk between SNF1-, cAMP-, TORC1-, and CWI-signaling (including the role of Rho5) has also been suggested by studies on yeast cells in stationary phase and their effect on chronological life span [24].

Mammalian cells not only carry Rac1 as a homolog of yeast Rho5, but also homologs of the subunits of the dimeric Dck1/Lmo1 GEF, namely different isoforms of DOCK and ELMO, which represent a small family of proteins forming dimeric GEFs for Rac1 [25]. The latter were first described for their role in the organization of the actin cytoskeleton [26,27], but are also involved in signaling of the blood glucose concentration in muscles and neurons [28,29]. Given these diverse functions, deregulation of Rac1 activity was found to trigger a number of diseases, such as breast cancer, diabetes, and neurological disorders [30,31,32].

Here we found that yeast Rho5 is also related to glucose signaling. Thus, a rapid relocation of Rho5 from the plasma membrane to mitochondria, along with Dck1/Lmo1, is triggered by glucose starvation. Synthetic phenotypes between deletions in genes encoding Rho5 or its activating GEF subunits with mutants defective in nutrient signaling components further supported a function of Rho5 in monitoring the cells nutrition state. In contrast to the function of its homolog Rac1 in mammalian systems, yeast Rho5 does not seem to play a major role in the organization of the actin cytoskeleton.

## 2. Results

### 2.1. Dck1 Forms Transient Foci in the Cytoplasm and Rapidly Accumulates at Mitochondria upon Glucose Starvation

**Figure 2:**
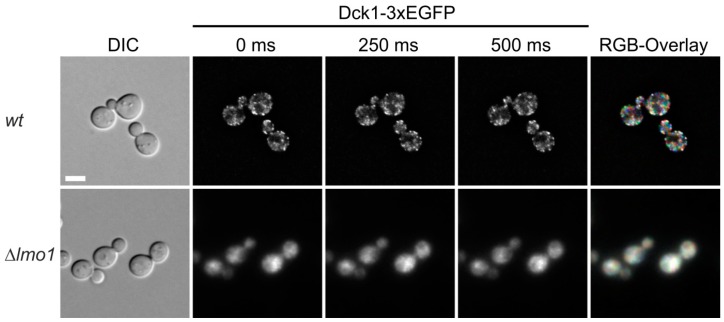
Dck1 forms transient foci. Images of individual cells forming foci were taken and color coded in the RGB overlay from different time-points to visualize movement (see also movie in supplementary materials). The diffuse distribution in the *lmo1* deletion was used as a control. The scale bar in the upper left image represents 5 µm and is valid for all images. See Materials and Methods for further details.

We previously found that Rho5 is associated with the plasma membrane under normal growth conditions and rapidly switches to mitochondria under oxidative stress, the latter being dependent on its dimeric GEF Dck1/Lmo1 [10]. This is in contrast to Dck1 and Lmo1 which show a diffuse cytoplasmic distribution in the absence of oxidative stress, with the occasional formation of foci in which the two proteins colocalize (Figure 1B and [10]). We wondered whether these foci are stable and investigated a relation to certain growth conditions, with a focus on glucose availability. For this purpose, a functional Dck1-3xEGFP fusion was employed for in vivo fluorescence microscopy. Cells grown in synthetic medium with 2% glucose displayed a number of foci, which appeared only in a subset of cells and changed their position in less than 250 ms (Figure 2; also see movie in Supplementary Material). Confirming our previous data, the foci only formed in the presence of Lmo1, reflecting the interdependence of the two GEF subunits. We previously showed that they do not colocalize with any known organelles or vesicles [10], and therefore speculate that the foci form as a transient Dck1/Lmo1 complex in response to subtle changes in intracellular conditions, which ultimately serve to transport active Rho5 to mitochondria.

**Figure 3:**
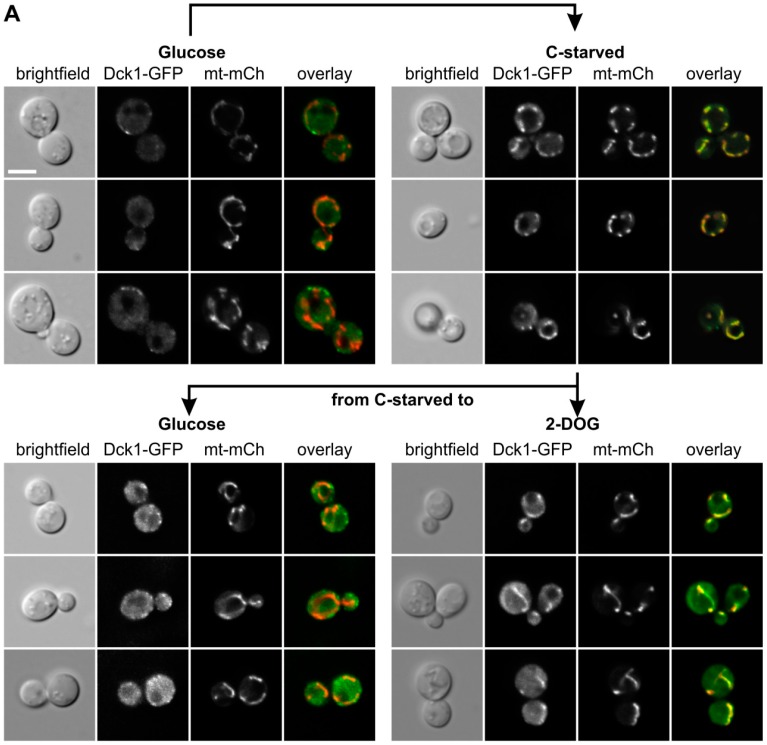

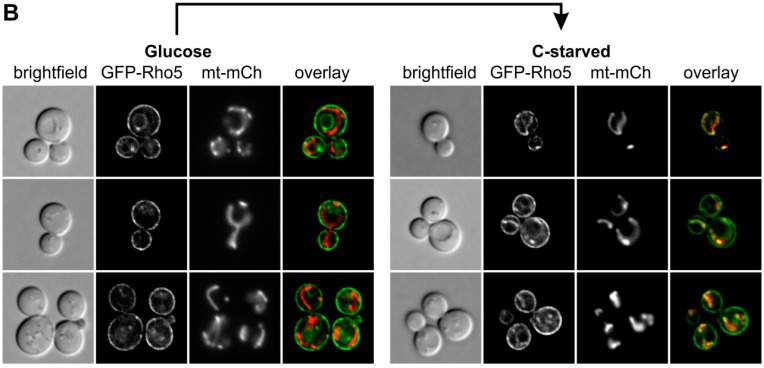
Dck1 and Rho5 rapidly accumulate at mitochondria upon glucose starvation. (**A**) Colocalization of Dck1-3×EGFP with mitochondria (mt-mCherry). Cells were starved for glucose (upper right) and either glucose or 2-deoxyglucose were added (lower panels). (**B**) Colocalization of GFP-Rho5 with mitochondria (*IDP1*-mCherry). The scale bar = 5 µm is valid for all images shown. Further details on strains and conditions used can be found in Materials and Methods.

Next, we exposed the cells to glucose starvation, by transfer to a medium without carbon source (Figure 3A). Fluorescence microscopy and Z-stacks of a strain carrying Dck1-3×EGFP demonstrated that a significant amount of the protein relocated to the mitochondria upon starvation, similar to the previously observed behavior under oxidative stress. Addition of glucose to such starved cells resulted in a rapid re-establishment of the diffused and patchy cytoplasmic distribution (Figure 3A, middle panel, left). To demonstrate that the glucose-dependent relocation in fact depends on glucose metabolism, we employed the non-metabolizable analog 2-deoxyglucose, and found that the mitochondria-associated localizaton of Dck1-3×EGFP is retained (Figure 3A, middle panel, right). To confirm that this mitochondrial switch is not confined to Dck1, we also examined the distribution of a GFP-Rho5 fusion protein under glucose starvation. As shown in Figure 3B, a significant proportion of Rho5 also relocated from the plasma membrane to mitochondria.

### 2.2. Rho5 Deletions Genetically Interact with Gpr1, Gpa2, and Sch9

**Figure 4:**
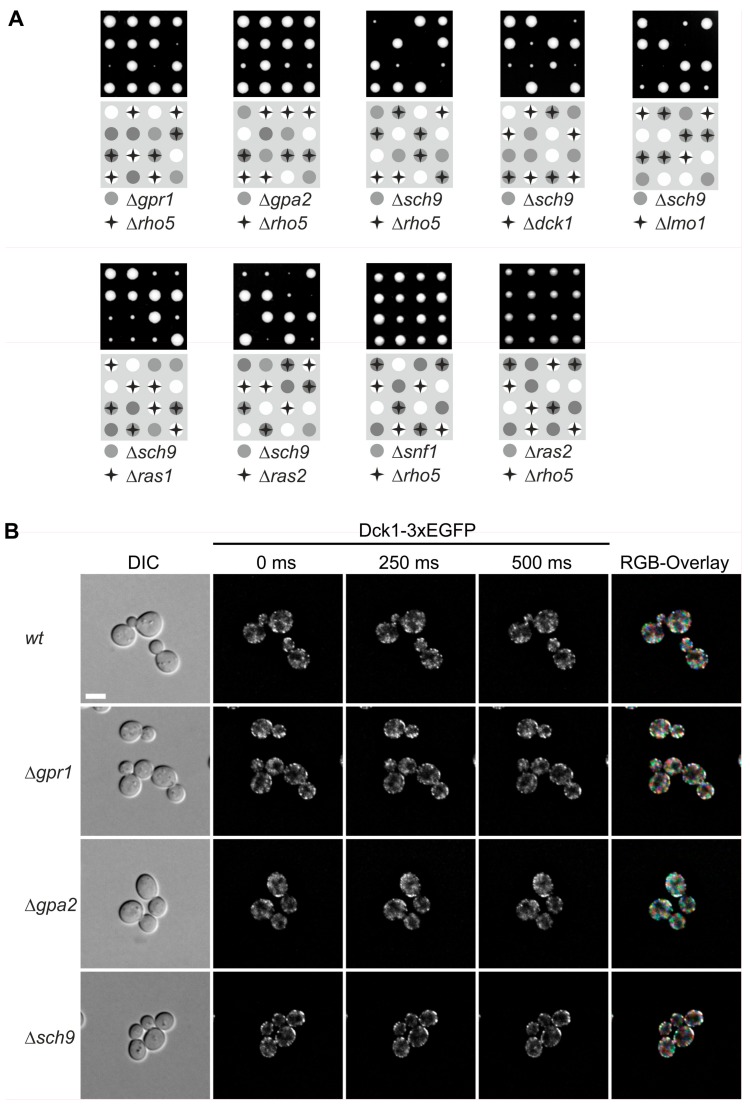
Deletions in genes encoding Rho5 or its dimeric GEF genetically interact with mutants in nutrient signaling, which is independent of foci formation. (**A**) Tetrad analyses reveal synthetic phenotypes between *rho5* deletions and mutants defective in glucose signaling. Tetrad analyses of strains carrying the heterozygous deletions as indicated were performed. Four representative tetrads carrying all combinations as coded in the lower panels are shown in each case. (**B**) Fluorescence microscopy of Dck1-3×EGFP foci in different signaling mutants. Images were taken at different times and color coded as described in Figure 2. For ease of comparisons, the wild-type images from Figure 2 have been included, again. The size bar in the upper left image represents 5 µm and is valid for all images shown. See Materials and Methods for further details.

Given the carbon source-dependent localization, we wondered whether any of the pathways monitoring glucose in the medium may interact with Rho5 and/or its GEF. Therefore, epistasis analyses were performed by crossing *rho5* deletion strains with different mutants impaired in glucose signaling. In view of the previously detected relation of Rho5 to autophagy and mitophagy [9,10], and the reported synthetic lethality of *gpr1* and *gpa2* with *sch9* (https://www.yeastgenome.org/; accessed on 20 June 2018), we also included the latter deletion (see Figure 1A for proposed interactions). Isogenic diploid strains being heterozygous for both *rho5* and either of these deletions were subjected to tetrad analyses. As evident from Figure 4A, both *gpr1* and *gpa2* showed a synthetic sickness with *rho5*, i.e., segregants carrying the respective double deletions produced much smaller colonies than those being wild-type or carrying either one of the deletion alleles. This phenotype appeared more pronounced for *gpr1 rho5* than for *gpa2 rho5*. On the other hand, a *sch9* deletion, which by itself displayed a drastic growth defect, was synthetically lethal with *rho5*, i.e., no viable progeny carrying the double deletion were obtained. Very similar synthetic phenotypes were also observed in combinations of *sch9* with either *dck1* or *lmo1* deletions, i.e., synthetic lethality of *dck1 sch9* and very poor growth of *lmo1 sch9* (Figure 4A). This demonstrated that an active Rho5 is of central importance for growth in the absence of Sch9.

Synthetic lethality or synthetic sickness was also observed for *ras2 sch9* double deletions (Figure 4A), whereas *ras2 rho5* double deletions were perfectly viable. No synthetic phenotype was observed in tetrad analysis of a diploid being heterozygous for both a *rho5* and a *snf1* deletion, indicating that SNF1-mediated glucose signaling is independent of Rho5 function (Figure 4A).

We wondered whether the genetic interactions observed above are also reflected in a physical interaction in living cells. To address this question, the distribution of a Dck1-3×EGFP fusion protein was analyzed in different deletion backgrounds as compared to a wild-type strain. Neither the diffuse distribution, nor the number and formation of patch-like structures, appeared to be notably altered in the null mutants (Figure 4B).

### 2.3. Deletions of RHO5, GPR1, GPA2, or RAS2 Result in Hyper-Resistance towards Cell Wall Stress Agents

**Figure 5:**
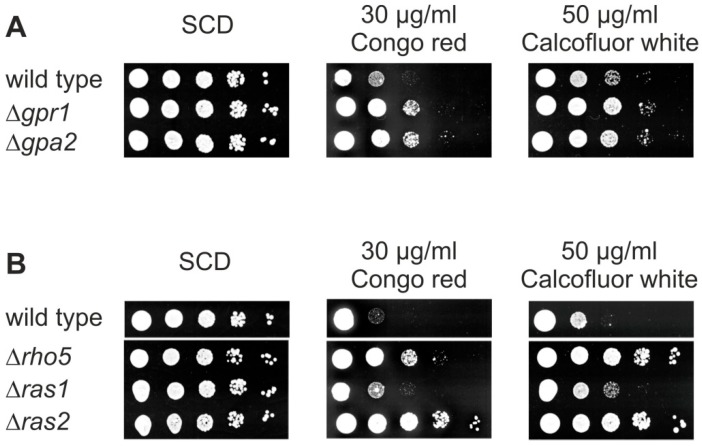
The lack of either Rho5, Ras2, Gpa2, or Gpr1 cause hyper-resistance towards cell wall stress agents. (**A**,**B**) Ten-fold drop dilution assays, from left to right, were performed on the media as indicated with haploid deletion strains as indicated. Strains used are described in Materials and Methods and in Table 1.

Since one of the first roles described for Rho5 was the negative regulation of CWI signaling [4], and in view of the synthetic phenotypes observed above, we wondered whether *S. cerevisiae* cells lacking other small GTPases may react to cell wall stress similar to a *rho5* deletion. We therefore performed drop dilution assays of deletion mutants on media containing Calcofluor white and Congo red. As evident from Figure 5A, strains lacking either the glucose sensor Gpr1 or its target G-protein Gpa2 [33] showed hyper-resistance towards the cell wall stress agents. Moreover, the *ras2* deletion displayed a strong hyper-resistance on these media, similar, or even stronger than, the one observed for the *rho5* deletion (Figure 5B). In contrast, the *ras1* deletion displayed only a moderate increase in resistance towards the cell wall stress agents. Note that mutants in the independent SNF1 signaling pathway were previously shown to be hyper-sensitive to the same agents and thus not included in these assays [13]. Taken together, these data further support a crosstalk of CWI and specific branches of the glucose signaling pathways, which could be mediated by Rho5.

### 2.4. Lack of Dck1 Moderately Affects Actin Dynamics in Budding Yeast Cells

**Figure 6:**
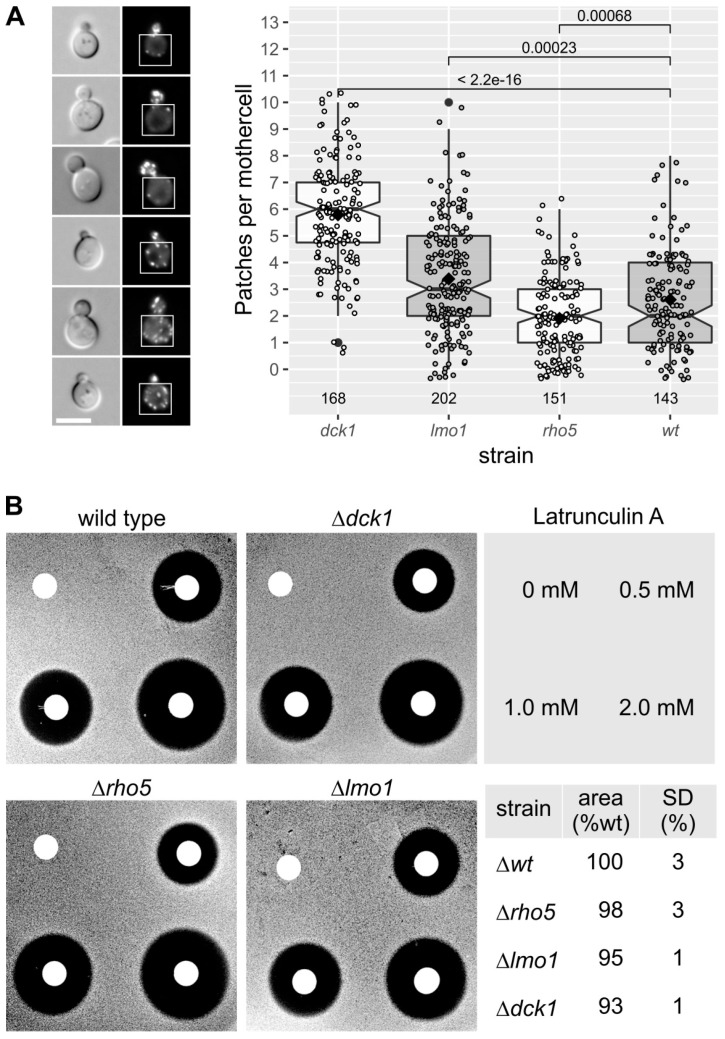
Deletion of *DCK1* moderately affects the actin cytoskeleton. (**A**) Life-cell fluorescence microscopy of strains carrying a genomic *CAP2-GFP* fusion in combination with the different gene deletions indicated and statistical analysis of actin patch numbers in mother cells. Exemplary images of cells with different numbers of actin patches in the mother cell are shown at the left. The scale bar is 5 µm. (**B**) Halo assay for Latrunculin A sensitivity. Further details on the interpretation of the box plot and the halo assay can be found in Materials and Methods.

One of the first roles of Rac1 and its dimeric GEF postulated in mammalian cells was its influence on the actin cytoskeleton [26,27,34]. In order to address the question whether this function may also be conserved in the yeast homolog we examined the different mutants lacking either one of the GEF subunits or Rho5 itself. A first hint was provided by an apparent increase in actin patches observed in the mother cells of a *dck1* deletion strain stained with rhodamine phalloidin. Since yeast cells grow primarily by deposition of new membrane and cell wall material at the bud tip, which depends on a careful balance between exo- and endocytosis, sites of endocytosis marked by actin patches are predominantly found in the growing bud and rarely in the larger mother cell [35,36]. To investigate whether Rho5 and its activating GEF play a role in actin dynamics, we employed a Cap2-GFP fusion for life-cell imaging of actin patches in deletion mutants lacking either of the three proteins (Figure 6A). While wild-type mother cells with small buds displayed only an average of two actin patches as expected, we found that *dck1* deletions showed a three-fold increase. A very moderate, but statistically significant, increase to an average of three patches per mother cell was also observed for the *lmo1* deletion, while the *rho5* mutant displayed a slight decrease as compared to wild-type in mother cells.

These results indicated that Rho5 activity does not strongly (and only indirectly) affect the actin cytoskeleton. To confirm these findings, sensitivity to Latrunculin A was tested for the three deletion mutants in comparison to the wild-type (Figure 6B). Perturbations of the actin cytoskeleton frequently lead to hyper-sensitivity towards this inhibitor of action polymerization [35,37]. A very moderate increase in resistance as compared to the wild type was observed for all deletions, which was most pronounced in the *dck1* strain, in which the area of the inhibition zone was reduced by an average of 7% as compared to wild type. Taken together, these data indicate that the major role of Rac1 and its dimeric GEF in controlling mammalian actin dynamics is only weakly conserved in its yeast homologs.

## 3. Discussion

As stated in the introduction, Rac1, the mammalian homolog of yeast Rho5, has a prominent function in organizing the actin cytoskeleton. Based on the results presented above, this function appears to be only marginally conserved in Rho5 of *S. cerevisiae*. Thus, strains lacking Rho5 showed neither a prominent effect on the low number of actin patches observed in yeast mother cells, nor on the sensitivity towards the actin-polymerization inhibitor Latrunculin A. The finding that actin patch numbers increased threefold in the *dck1* deletion as compared to wild-type mother cells, and 30% in the *lmo1* deletion, indicates that either the dimeric GEF Dck1/Lmo1, or Dck1 by itself, may interact with another GTPase which is involved in the organization of the actin cytoskeleton. Cdc42, Rho2, or Rho3 would be possible candidates for such an additional weak interaction [38]. Moreover, Dck1 is occasionally associated with the yeast bud neck, indicating that it could interact with the actomyosin ring during cytokinesis (Figure 1B). While such a function has been postulated for the Rho5 homolog in the related filamentous fungus *Ashbya gossypii* [39], the lack of morphological defects in the yeast *rho5* deletion suggests that such an interaction, if it exists, would only have a weak physiological effect. Interestingly, *dck1* and *lmo1* deletions were slightly more resistant, rather than more sensitive, towards Latrunculin A. One possible explanation for this phenotype could be the observed increase in actin patches, which may reflect an increase in polymerization activity, thus counteracting the effect of the inhibitor to some extent. A relation of Rho5 to actin organization was also suggested by previous findings, in which depolarization of the cytoskeleton upon heat shock was abolished in the *rho5* deletion, but enhanced by a hyper-active *RHO5* allele [4]. In that work, the effect was attributed to the role of Rho5 as a negative regulator of CWI signaling. Taken together with the data presented here, we conclude that the influence of Rho5 on actin organization in *S. cerevisiae* is probably indirect, may be limited to secondary interactions with its dimeric GEF, and is not essential under the growth conditions tested.

Instead, our data indicate that Rho5 may be implicated in the response to glucose starvation. This adds yet another important physiological function to what has already been reported for its role in CWI signaling [4], the responses to high medium osmolarity (HOG pathway [6]) and oxidative stress, including the consequences for mitophagy and apoptosis [9,10], and the maintenance of chronological lifespan [24]. The notion that Rho5 fulfils the role of a central hub that integrates signals from different stress response and survival pathways [10] is further supported by the new findings presented herein. For single-cell organisms like yeast, availability of nutrients, especially of glucose as a preferred carbon source, needs to be carefully monitored and rapidly related to metabolic control and neutralization of harmful processes, such as perturbances in cytoplasmic pH homeostasis or mitochondrial production of reactive oxygen species (ROS; recently reviewed in a previous paper [22]).

More specifically, the availability of glucose in *S. cerevisiae* is sensed and converted to proper transcriptional and physiological responses by different routes, some converging in cAMP/PKA signaling like the Ras1/2 and the Gpr1/Gpa2 branches, and another important pathway which involves the SNF1 complex (Figure 1A, reviewed in previous papers [11,12,40]). Based on the genetic interactions found herein, Rho5 appears to mediate the crosstalk to the former route and does not affect SNF1 signaling. The fact that *rho5* deletions are synthetically lethal or synthetically sick with *gpr1* or *gpa2* deletions indicates that Rho5 acts in parallel to these glucose sensing components upstream of a common effector. In addition, *rho5* also genetically interacts with *sch9*. What could be the common denominator of all these findings? The Sch9 protein kinase is a downstream effector of nutrient signaling by TORC1, which primarily monitors nitrogen availability [41], but has also been implicated in glucose signaling [11,22]. If glucose is available, signaling by Sch9 and cAMP/PKA, sequester the Rim15 kinase in the cytoplasm (Figure 1A). Consequently, Msn2/4, the central transcription factors which respond to general stress conditions like nutrient starvation and oxidative stress [42,43], cannot be phosphorylated and are exported from the nucleus. It is tempting to speculate that Rho5 associated with the plasma membrane could play a similar role in a parallel glucose-responsive pathway, i.e., in contributing to sequestration of Rim15 in the cytoplasm. The observed switch of the Rho5/Dck1/Lmo1 complex to mitochondria upon glucose starvation would then relieve this regulation. As a result, Rim15 and Msn2/4 can trigger the responses to sugar depletion and other stresses.

Similar to *gpr1* or *gpa2*, a *ras2* deletion is synthetically lethal with *sch9*, and *gpr1*, *gpa2*, and *ras2* deletions share the hyper-resistance of a *rho5* deletion towards cell wall stress agents. The lack of synthetic lethality between *rho5* and *ras2* appears somewhat surprising in the model derived from these observations and proposed in Figure 1A. However, it can be explained by a variety of redundant functions: (i) Ras1 activity could be sufficient in this context to compensate the lack of Ras2. (ii) In the absence of Ras2 Gpr1/Gpa2 activity could provide sufficient cAMP/PKA signaling to cope with carbon stress conditions, as long as Rho5 is intact, whereas Ras2 activity would not be sufficient in the absence of Gpr1/Gpa2. (iii) Gpr1/Gpa2 may serve an additional, yet unknown function independent of cAMP/PKA signaling that requires Rho5 activity.

In addition to the rather slow transcriptional adaptations just discussed, intracellular changes provoked by sudden changes in glucose availability also require much more rapid responses. In this context, Rho5 and its activating GEF are of special interest. Thus, Dck1 and Lmo1 can form granules in the yeast cytoplasm, which do not colocalize with any known vesicular or membrane structures [10]. Granule formation is interdependent, i.e., prevented by loss of either Dck1 or Lmo1. More importantly, such strains also fail to relocate Rho5 from the plasma membrane to mitochondria upon exposure of cells to oxidative stress [10]. While the exact intracellular cues leading to formation of the granules remain to be defined, once formed they are highly mobile (Figure 2), which probably accounts for the rapid transfer of Rho5 to mitochondria. The fact that GFP-Rho5 is either located at the plasma membrane or at the mitochondrial surface, but cannot be observed in the granules, indicates that only a small fraction of cytoplasmic Dck1/Lmo1 patches is loaded with Rho5 at any given time point and the GTPase is rapidly released at its target site.

In addition to the previous reports, herein we showed that the rapid switch to mitochondria is also provoked by carbon starvation, implicating Rho5 in the control of the transition from fermentative sugar utilization by glycolytic enzymes in the cytoplasm to respiratory energy production in mitochondria. This relocation is (i) rapid and (ii) reversible, since Rho5 does no longer accumulate at mitochondria, when glucose is added to starved cells. Clearly, this is not merely an indirect effect, since addition of the non-metabolizable glucose analog 2-DOG cannot reverse mitochondrial localization of Rho5 or Dck1 in starved cells. The putative function of this switch in triggering mitophagy would further reduce the physiological damage caused by increased ROS formation linked to respiratory processes [10].

On a broader scale, growing evidence implicates Rac1 and its GEF in mammalian cells in the control of central carbohydrate metabolism and disease development [28,29,31,32]. The findings presented herein pave the ground for detailed studies on the molecular regulatory circuits underlying these phenotypic effects in eukaryotic cells, once again mining the power of yeast genetics as a valuable tool. Moreover, given the higher similarity in the physiology of mammalian cells to non-fermentative yeast species, future research may also focus on the role of the Rho5 complex in the Crabtree-negative model yeast *Kluyveromyces lactis* [44,45]. Preliminary data from our laboratory in fact indicate that Rac1 functions are more strongly conserved in its KlRho5 homolog than in Rho5 from *S. cerevisiae*.

## 4. Materials and Methods

### 4.1. Strains and Growth Conditions

Yeast strains employed and their genotypes are listed in Table 1. In brief, all strains were isogenic except for the mutant alleles indicated and derived from HD56-5A and its isogenic diploid DHD5 [46,47], which also constitutes one of the parental strains of the common CEN.PK series [48]. Yeast cell culture and genetic techniques followed standard procedures [49]. Rich medium (YEPD) contained 1% yeast extract, 2% Bacto peptone (both by Becton, Dickinson and Company, Sparks, MD, USA), and 2% glucose. Synthetic media were prepared as described previously from yeast nitrogen base (Becton, Dickinson and Company, Sparks, MD, USA) [49], with the omission of amino acids or bases as required for selection of plasmids or deletion markers and 2% glucose (SCD; alpha-d-Glucose from Serva Electrophoresis GmbH, Heidelberg, Germany) as carbon source. Selection for the *kanMX* marker was achieved by addition of 200 mg/L of G418 (g418 sulfate purchased from Carl Roth GmbH and CoKG, Karlsruhe, Germany). The Latrunculin A (purchased from Sigma-Aldrich Chemie GmbH, Munich, Germany) halo assay was performed basically as described previously [37]. In short, YEPD plates with 30 mL medium, each, were prepared. Top-layer agar was prepared with YEPD containing 1% agar, aliquoted in 4 mL glass tubes and kept at 48 °C. Yeast strains were grown to late logarithmic phase overnight in 2.5 mL YEPD, each diluted 1:10 in fresh YEPD and 400 µL, each sample was inoculated in the top-layer, mixed and poured onto the YEPD plates. Sterile filter discs were prepared and soaked with 10 µL each of either DMSO or stock solutions of Latrunculin A at the given concentrations. Filter discs were placed on the plates, which were then incubated over night at 30 °C. Plates were scanned with and without a scaled background and used to determine the size of the halos. Strains used in Figure 6B were: HOD294.2-1A (wild-type), HOD294.2-1B (wild-type), HOD294.2-2B (*Δrho5*), HOD294.2-3B (*Δrho5*), HOD314-5A (*Δdck1*), HOD314-8A (*Δdck1*), HAJ201-A (*Δlmo1*), HAJ201-B (*Δlmo1*), and HMZ18-A (*Δlmo1*). The assay was performed twice for each of the nine strains, using 0, 0.1, 0.5, and 1.0 mM of Latrunculin A in the first assay and the concentrations indicated at the upper right in the second assay. Images show one example of each mutant type from the second assay. The areas of all inhibition zones (i.e., a minimum of 10 halos for each mutant type) were determined as described in materials and methods, averaged, and expressed as percentage of wild-type, including the standard deviations (SD; lower right hand).

For serial drop-dilution assays cells were grown over night in SCD to late logarithmic phase, diluted in fresh medium to a final OD_600_ of 0.3, and subjected to serial 10-fold dilutions. Three µL of each dilution was spotted onto the media as indicated and plates were incubated for 3 days at 30 °C. Images were scanned and adjusted for brightness and contrast using Corel Photo Paint with the same settings for the entire plate, prior to compilation of lanes into the final figures. Strains employed in Figure 5A were HAJ6-A (wild-type), HAJ187-A (*Δgpr1*), and HAJ188-A (*Δgpa2*). Strains employed in Figure 5B were HAJ6-A (wild-type), HAJ216-A (*Δrho5*), HAJ217-A (*Δras1*), and HAJ218-A (*Δras2*).

For tetrad analyses, diploid strains were grown to stationary phase in liquid YEPD, collected by centrifugation, and dropped onto 1% potassium acetate agar for sporulation at 30 °C. After 2–4 days and microscopic inspection for ascus formation, a sample of each culture was resuspended in 100 µL of sterile water and 4 µL of Zymolyase 20T (10 mg/mL; MP Biomedicals, Eschwege, Germany) were added. After incubation for 7–10 min at room temperature 10 µL of the suspension was streaked out onto a YEPD plate and spores were segregated using a Singer MSM400 micromanipulator (Singer Instruments, Somerset, UK). Plates were incubated for 3 days at 30 °C and colony formation was documented by scanning and image processing as described for the serial drop-dilution assays.

For manipulations in *E. coli*, strain DH5α was employed with media, as described previously [51].

### 4.2. Construction of Plasmids, Deletion Mutants and Gene Tagging

All strains used herein were modified at their original loci using one-step gene replacement techniques [52]. Deletions were obtained with either the *kanMX* or the *SkHIS3* cassette from the pFA6a series of the Longtine collection [53] (note that this collection contains the *HIS3* gene from *Saccharomyces kluyveri* instead of the *HIS5* gene from *Schizosaccharomyces pombe*; in strains carrying the latter deletion marker, it was obtained from pUG27 [54]). For replacement of deletion markers by in vivo recombination, the plasmid pJJH1286 was constructed, which carries the *KlURA3* gene flanked by the TEF promoter and terminator regions of the pFA6a series. The 3xEGFP fluorescence marker was originally obtained from a plasmid described in [55]. Plasmid pJJH1408 (*CEN/ARS*, *LEU2*, mt-mCherry) was introduced for colocalization of GFP fusions with mitochondria, as described previously [10]. Alternatively, a genomic fusion of the *IDP1* gene with the coding sequence for mCherry was constructed using the oligonucleotide pair 15.254/15.255; 5′-ATGCCGTTGAAAAAAGACTACAAAAAGAAATCAAGTCGATCGAGCGGATCCCCGGGTTAATTAA-3′/5′-AAAAAAAAGTAGTTCATTATCCTAGAGCATAACTAAGTATAAGAATTCGAGCTCGTTTAAAC-3′ with plasmid pJJH1525 as a template (a pFA6a-SkHIS3 derivative constructed herein) to generate a PCR-tagging cassette, and employed for colocalization with mitochondria. Plasmid pJJH1639 (*CEN/ARS*, *LEU2*, *GFP-RHO5*) was constructed by in vivo recombination to obtain an N-terminal GFP fusion with the coding sequence of *RHO5*. Sequences of the plasmids and modified loci and further details on constructions and oligonucleotides used are available upon request.

### 4.3. Strains and Experimental Conditions for Fluorescence Microscopy, Image Acquisition and Statistical Analyses

The setup used for fluorescence microscopy consisted of a Zeiss Axioplan 2 (Carl Zeiss, Jena, Germany) equipped with a 100× alpha-Plan Fluor objective (NA 1.45) and differential-interference contrast. Sample handling and image processing have been described previously [10].

In Figure 2, cells of strain HAJ152-A (Dck1-3×EGFP) were grown to early logarithmic phase in synthetic minimal medium containing glucose, transferred to fresh medium, and allowed to grow for 3–4 h at 30 °C. Images of individual cells forming foci were taken and color coded at different time-points to visualize movement in the RGB overlay. Strain HAJ207A, carrying an *lmo1* deletion, was employed as a control, confirming the previously observed lack of foci formation [10].

In Figure 3, Strain HAJ152-A producing Dck1-3×EGFP (designated in the figure as Dck1-GFP) was investigated by fluorescence microscopy. For these investigations a plasmid with a mCherry fusion to a mitochondrial signaling sequence (mt-mCH; pJJH1408) was also introduced into strain HAJ152-A to allow for colocalization. Cells were grown in SC medium with 2% glucose and re-inoculated for 3–4 h, as described above, for Figure 2, washed twice with SC medium without carbon source, and resuspended in this medium for carbon starvation (C-starved). Cells were observed under the microscope between 5–15 min after the transfer (upper panels). After 30 min of starvation cells were collected by centrifugation and resuspended in SC containing either 2% glucose (lower left) or 2-deoxyglucose (lower right), incubated for 10 min, and again subjected to fluorescence microscopy. Images taken were, from left to right: bright field, GFP channel, mCherry channel, and overlay. Three representative images are shown for each condition.

In Figure 4, tetrad analyses of strains carrying the heterozygous deletions as indicated were performed for at least ten tetrads for each cross, confirming the synthetic phenotypes shown in four representative tetrads in the figure. Tetrads were separated on YEPD plates with 2% glucose by micromanipulation and segregants were allowed to grow for 3 days at 30 °C (upper panel). Note that the strain heterozygous for the *snf1* deletion was segregated on YEPD with 5% glucose to avoid the slow-growth phenotype of this null mutant [13]. The lower panels show schematic representations with filled circles and stars indicating the combination of mutant alleles for each segregant; white circles represent wild-types. Strains employed were: DAJ145 (*Δrho5/RHO5 Δgpr1/GPR1*), DAJ144 (*Δrho5/RHO5 Δgpa2/GPA2*), DAJ138 (*Δrho5/RHO5 Δsch9/SCH9*), DAJ119 (*Δdck1/DCK1 Δsch9/SCH9*), DAJ128 (*Δlmo1/LMO1 Δsch9/SCH9*), DAJ139 (*Δras1/RAS1 Δsch9/SCH9*), DAJ140 (*Δras2/RAS2 Δsch9/SCH9*), HOD294.2 (*Δrho5/RHO5 Δsnf1/SNF1*), and HOD320 (*Δrho5/RHO5 Δras2/RAS2*). For fluorescence microscopy, images were taken at different times and color coded as described in Figure 2. Strains employed were: HAJ204-A (*Δgpr1 DCK1-3×EGFP*), HAJ205-A (*Δgpa2 DCK1-3×EGFP*), and HAJ206-A (*Δsch9 DCK1-3×EGFP*).

In Figure 6A, strains carrying a genomic *CAP2-GFP* fusion were used in combination with different gene deletions: HOD310-1B (wild-type *CAP2-EGFP*), HOD309-1D (*Δrho5 CAP2-EGFP*), HOD314-2C (*Δdck1 CAP2-EGFP*), and HOD310-4A (*Δlmo1 CAP2-EGFP*). Cells were grown to early logarithmic phase in synthetic glucose medium and actin patches were observed by fluorescence microscopy. Mother cells with small to medium size buds were selected manually and the number of actin patches was determined by thresholding in combination with the “Analyze particles” function of the ImageJ software (ImageJ 2.0.0-rc-65/1.51s, [56]). Exemplary images of cells with different numbers of actin patches in the mother cell are shown at the left, with brightfield-images (left) and fluorescence images showing GFP signals (right). White boxes indicate the area used for quantification of patches. The boxplot on the right gives the results of this analysis for the number of mother cells indicated by the numbers below for each strain. The lower and upper hinges correspond to the first and third quartiles (the 25th and 75th percentiles). Whiskers mark values which are within 1.5 times the interquartile range. The notches give a roughly 95% confidence interval comparing medians. Means are represented by a black diamond. The number of cells analyzed for each strain is given above the deletion allele name (wt = wild-type for the genes in question). In addition, *p*-values of pairwise ANOVA tests for each mutant as compared to the wild type are given above.

Automated counting of actin patches and measurement of the halo-areas in the Latrunculin A assay were performed by automatic thresholding in combination with the “Analyze particles” function of the ImageJ distribution Fiji (ImageJ 2.0.0-rc-65/1.51s, [56]).

Statistical analysis and plots were made using R version 3.5.0 [57,58] (R Core Team 2018) in combination with Rstudio desktop version 1.1.453 [59], with modules ggplot2, plyr, and dplyr [60,61].

## Figures and Tables

**Table 1:** Strains used in this study.

Strain	Genotype	Source
DHD5	*MATa/α ura3-52/ura3-52 leu2-3,112/leu2-3,112 his3-11,15/his3-11,15*	[47]
HAJ6-A	*MATa* segregant from DHD5	[50]
HAJ6-B	*MATα* segregant from DHD5	[50]
DAJ119	as DHD5 except *dck1::kanMX/DCK1 sch9::SkHIS3/SCH9*	This study
DAJ128	as DHD5 except *lmo1::kanMX/LMO1 sch9::SkHIS3/SCH9*	This study
DAJ138	as DHD5 except *rho5::KanMX/RHO5 sch9::SkHIS3/SCH9*	This study
DAJ139	as DHD5 except *ras1::KanMX /RAS1 sch9::SkHIS3/SCH9*	This study
DAJ140	as DHD5 except *ras2::KanMX /RAS2 sch9::SkHIS3/SCH9*	This study
DAJ144	as DHD5 except *rho5::KanMX/RHO5 gpa2::KanMX/GPA2*	This study
DAJ145	as DHD5 except *rho5::KanMX/RHO5 gpr1::KanMX/GPR1*	This study
HAJ152-A	as HAJ6-A except *DCK1-3xmyEGFP::SpHIS5*	[10]
HAJ187-A	as HAJ6-A except *gpr1::KanMX*	This study
HAJ188-A	as HAJ6-A except *gpa2::KanMX*	This study
HAJ201-A	as HAJ6-A except *lmo1::KanMX*	This study
HAJ201-B	as HAJ6-B except *lmo1::KanMX*	This study
HAJ204-A	as HAJ6-A except *gpr1::KanMX DCK1-3xEGFP::SpHIS5*	This study
HAJ205-A	as HAJ6-A except *gpa2::KanMX DCK1-3xEGFP::SpHIS5*	This study
HAJ206-A	as HAJ6-A except *sch9::KanMX DCK1-3xEGFP::SpHIS5*	This study
HAJ207-A	as HAJ6-A except *lmo1::KanMX DCK1-3xEGFP::SpHIS5*	This study
HAJ216-A	as HAJ6-B except *rho5::KanMX*	[10]
HAJ217-A	as HAJ6-A except *ras1::KanMX*	This study
HAJ218-A	as HAJ6-A except *ras2::KanMX*	This study
HCS076-A	as HAJ6-A except *rho5::KanMX IDP1-mCherry::SkHIS3*	This study
HMZ18-A	as HAJ6-A except *lmo1::SkHIS3*	This study
HOD294.2	as DHD5 except *rho5::kanMX/RHO5 snf1::SpHIS5/SNF1*	This study
HOD320	as DHD5 except *rho5::kanMX/RHO5 ras2::SkHIS3/RAS2*	This study
HOD294.2-1A	as HAJ6-A	This study
HOD294.2-1B	as HAJ6-B	This study
HOD294.2-2B	as HAJ6-B except *rho5::KanMX*	This study
HOD294.2-3B	as HAJ6-A except *rho5::KanMX*	This study
HOD309-1D	as HAJ6-A except *rho5::KanMX CAP2-EGFP::SpHIS5*	This study
HOD310-1B	as HAJ6-A except *CAP2-EGFP::SpHIS5*	This study
HOD310-4A	as HAJ6-A except *lmo1::SkHIS3 CAP2-EGFP::SpHIS5*	This study
HOD314-2C	as HAJ6-A except *dck1::KlURA3 CAP2-EGFP::SpHIS5*	This study
HOD314-5A	as HAJ6-A except *dck1::KlURA3*	This study
HOD314-8A	as HAJ6-B except *dck1::KlURA3*	This study

## Supplementary Materials

Supplementary materials can be found at http://www.mdpi.com/1422-0067/19/8/2186/s1.

## Abbreviations

AMPK	5′ adenosine monophosphate activated kinase
2-DOG	2-deoxy glucose
GEF	GDP/GTP exchange factor
PKA	protein kinase A
ROS	reactive oxygen species
SNF	sucrose non-fermenters
TORC1	target of rapamycin complex 1
vATPase	vacuolar ATPase complex
